# Parental evaluation of teachers’ competences and characteristics during COVID-19 pandemic homeschooling

**DOI:** 10.1007/s11218-022-09701-x

**Published:** 2022-06-28

**Authors:** Verena Letzel, Marcela Pozas, Kris-Stephen Besa

**Affiliations:** 1 Universität Trier, Universitätsring 15, Raum 441, 54296 Trier, Germany; 2 Professional School of Education (PSE), Humboldt-Universität zu Berlin, Unter den Linden 6, Raum: 0206 (2.OG), 10099 Berlin, Germany; 3 Professional School of Education (PSE), Humboldt-Universität zu Berlin, Sitz: Hausvogteiplatz 5-7, Raum: 0206 (2.OG), 10117 Berlin, Germany; 4 Universität Münser, Bispinghof 5/6 Raum F08, 48143 Münster, Germany

**Keywords:** Teachers’ digital competences, Differentiated instruction, Teacher feedback, Parental evaluations, Homeschooling

## Abstract

With the spread of the SARS-CoV-2 virus, school-related closures and the hasty transition into homeschooling, parents were required to take a more active and positive role than ever before in collaboration with their children’s educators. Thus, with this unprecedented situation, parents became an important source of information during the pandemic. Considering this unique event, the study at hand aims to explore parents’ perspectives regarding primary and upper secondary school teachers’ digital competence, digital differentiated instruction, and feedback during the first SARS-CoV-2 school shutdown in Germany. Additionally, the study examined parents’ evaluation of teachers’ characteristics. Results reveal that parental ratings were generally positive. Moreover, parents with children attending primary schools perceived and evaluated teachers’ digital competence and digital differentiated teaching highly, and perceived significantly more teacher feedback than parents whose children attend upper secondary school. In addition, positive correlations were found between parents’ evaluations of teachers’ feedback and how motivated, appreciative, and devoted teachers are perceived to be. Practical implications and further research areas are discussed.

## Introduction

Spring 2020 will be remembered as the time when the world found itself to be in a state of emergency (Education International, 2020; OECD, 2020). To prevent the rapid spread of the SARS-CoV-2 virus (COVID-19), policymakers worldwide decided to establish a lockdown on public life. An essential area that was severely affected by the lockdown situation was the educational sector. The establishment of emergency remote teaching (ERT, Bozkurt & Sharma 2020) created immense challenges for all stakeholders in the educational system (Dempsey & Burke, 2020; Education International, 2020; Carlson et al., 2020): schools were closed, teachers were rapidly forced to continue educating their students from home, and parents had, when possible, to work from home and support their children’s learning process at the same time. Thus, bestowed on teachers as well as parents was the responsibility of preventing their students/children from experiencing negative consequences as well as ensuring an effective online learning situation. With this background, parental participation and involvement became a crucial element for an effective implementation of ERT.

The implementation of ERT raised concerns that existing educational disparities would be acutely increased, having a critical impact on highly disadvantaged students (Gupta & Knaur Jawanda, 2020; UNESCO, 2020). Thus, the evaluation of teacher competence and characteristics has become even more important in order to identify teachers’ professional development needs in order to ensure a meaningful and high-quality instruction in such unprecedented times, as well as after the pandemic. Teacher evaluation systems make use of a manifold of approaches, such as educational stakeholders (i.e. students and parents), student test scores, and lesson observations, among others (Master, 2013). However, given the current educational situation, parents have become an important source of information concerning teachers’ competence and characteristics. Additionally, during ERT, “parents can be considered as one of the most important stakeholders of distance education, as they are the only ones who physically accompany their children” (Misirli & Ergulec, 2021, p. 6701). With this unique opportunity during which each single educational pathway was severely affected, the study at hand aims to explore parents’ perspectives regarding their children’s teachers during the first COVID-19 shutdown in Germany. In detail, the study examines parents’ evaluation of primary and upper secondary school teachers’ characteristics, such as motivation, flexibility, and fairness, as well as digital competences, differentiated instruction, and feedback towards students and parents. The following sections will briefly discuss the topics of professional teaching competence and teacher characteristics, incorporating the role of educational pathways as well as general measures of teacher evaluation and current research on parents’ perspectives during COVID-19 homeschooling.

## Teacher competence and ERT

Teachers should develop competences throughout the different phases of teacher training as well as during their in-service practice (Grangeat & Gray, 2007). International research has pointed to components of teacher competence and characteristics as the foundation for teaching quality and effectiveness that include, for instance, teacher-student relationships (Maulana et al., 2013), quality of the curriculum, efficient classroom management, quality of instruction (Kottler et al., 2005), teaching learning strategies, monitoring students’ progress (Witcher & Onwuegbuzie, 1999), and the adaptation of teaching to address students’ individual needs (Maulana et al., 2017; van de Grift, 2014). Moreover, besides developing professional competence, teachers should also build professional characteristics (McBer, 2001) such as fairness and commitment (Jahangiri & Mucciolo, 2008; Peng et al., 2014), as well as motivational orientations (Shulman, 1986). With the onset of the pandemic and the resulting school closures, the rapid and unexpected transition into ERT posed an unprecedent challenge for teachers (Carrillo & Flores, 2020; Kim & Asbury, 2020), and consequently, for their teaching competences and characteristics. Rice and Dawly (2009), however, explain that effective teaching practices and behaviors in classrooms do not always equate into effective teaching in an online environment. To this end, Mankki (2021) argues that emergency remote distance teaching requires teachers to modify their teaching practices and behaviors used in traditional in-school settings. With this context, the present study focuses on investigating crucial teaching behaviors and competences that are not only key factors within in-school learning (Hattie, 2009), but are also crucial for the development of ERT teaching and learning (Helm et al., 2021; König et al., 2020). The domains of teaching considered within this investigation are presented below.

### Digital competence

Since before the COVID-19 pandemic, teachers have been continuously expected to develop and teach lessons using digital tools and resources in order to support and maximize student learning. Worldwide, official teacher training standards (e.g. International Society for Technology in Education [United States of America], 2008; Standing Conference of the Ministers of Education and Culture [abbreviated “KMK” in German]) have aimed at fostering digital competences and thus have incorporated digital competences into their guidelines. For instance, in the case of Germany (the country in which this study was conducted), the Standing Conference of the Ministers of Education and Culture released a strategy paper in 2017, which was updated in 2021. This strategy paper comprises several fields of action such as constructing the legal and functional frameworks to establish learning management programs, as well as providing equipment and infrastructure to teachers, among others. However, despite these efforts, current international research findings show that teachers infrequently make use of digital tools and resources (Tondeur et al., 2013; Urez et al., 2018), and when they do, it appears that they do so mainly for administrative purposes rather than for teaching processes (Krause et al., 2017). Additionally, recent research conducted during the COVID-19 school related closures reveals that teachers face immense challenges when integrating digital instruments and tools into their teaching (Bozkurt & Sharma, 2020; Seufert et al., 2021). For example, a German study conducted by König et al. (2020) reported that the majority of teachers did not provide online lessons, with almost 70% of teachers not using any digital instruments to teach online, and only 20% of them conducted online assessments. Further analyses by König et al. (2020) showed that, as expected, teachers’ digital competences were related to how they mastered the challenges that came about during emergency remote education.

### Differentiated instruction

Along with digital competences of teachers and increased implementation of digitalization in the classroom, teachers are required to provide differentiated teaching materials addressing students’ individual needs (Graf, 2013; KMK, 2017). Effective teaching requires that teachers embrace diversity and address meaningfully the broad array of their students’ learning needs. It is especially this facet that plays an important role when it comes to the current ERT situation (Education Endowment Foundation, 2020). Students are required to learn at home without the structure and support they are used to receive in classroom teaching. Thus, it is necessary that teachers ensure, even during ERT, that “all children regardless of ability level are included in classrooms with their age-matched peers” (Sokal & Sharma, 2013, p. 59) and receive appropriate education (McCrimmon, 2015; Gray et al., 2017). However, the challenges imposed by the need for continuous professional development to meet the demands of diverse learners, which certainly increased during the pandemic, may have not been sufficiently addressed by teacher training programs and/or professional development courses (McCrimmon, 2015; Gray et al., 2017).

### Feedback

Another crucial component is feedback (Praetorius et al., 2018), not only between teachers and students, but between teachers and parents as well. According to Hattie and Timperley (2007) as well as Rahman et al. (2011), teachers’ feedback (also taking assessments into consideration) for students and their parents has a strong influence on student learning and achievement. In line with Siebert et al. (2018), feedback is understood in this study as teacher-student-parent communication (TSPC), which includes forms of summative and formative assessment and/or feedback (Sardareh, 2016; Tante, 2018). An adequate TSPC can increase school-related parental care and parental monitoring and involvement (Avvisati et al., 2013; Bergman, 2015). A recent study by Bubb and Jones (2020) exploring student, parent, and teacher experiences during the first COVID-19 lockdown showed that participants indicated that communication through digital tools and resources appeared to provide new opportunities for the exchange of feedback. In particular, teachers reported having more time to provide feedback, and interestingly, they indicated giving more useful feedback than usual. Moreover, parents also expressed positive feelings about the feedback provided by their children’s teachers.

### Teachers’ professional characteristics

Teaching effectiveness and quality is not only reflected by a teacher’s professional competence or instructional strategies. Many researchers emphasize the importance of more personal characteristics of teachers, such as flexibility, support, dedication, motivation, or their care for their students (Stronge, 2007; Tickle, 1999; Valenta, 2010). For instance, Miller (2012) points at students’ views of important personal teacher characteristics such as fairness, motivation, interest in the student, patience, self-reflectiveness and diligence. Research has shown that students’ perceptions of their teachers’ personal characteristics, for instance fairness, are associated with higher achievement outcomes (Peter et al., 2012). In the case of parents’ perspectives, a study by Liu and Meng (2009) found that parents who rated their children’s teachers as fair, caring, responsible, patient, and humorous respectively considered them “good teachers”. Recent research on ERT during the COVID-19 pandemic has shown that, although parents have reported that their children’s teachers have sought to provide support and have cared for their students, there was a lack of motivation, development of interesting classes, and development of online social contact from teachers’ side (Misirli & Ergulec, 2021). In addition to differences across personal characteristics, important variations can also be due to context factors, such as educational pathways.

### Educational pathways and teachers’ competence and characteristics

Research has shown that teaching behaviors differ between primary and upper secondary teachers (OECD, 2018), and that such an effect may result from the different teacher preparation programs and degrees across countries (Maulana et al., 2017; Rice, 2003). For instance, primary teacher education programs have a higher proportion of pedagogical content knowledge courses which include dealing with diversity, DI practices, and teaching methods, whereas upper secondary school teacher programs focus more on subject knowledge (OECD, 2021). Additionally, findings from TALIS have shown that primary and upper secondary teachers have different reasons for becoming teachers in the first place. For instance, upper secondary teachers report factors related to job security and reliable income as the most prevalent reasons to join the profession, whereas primary teachers cite aspects of altruistic motives as their main motivation (OECD, 2019).

Research exploring differences between educational pathways (primary and upper secondary) have shown that primary school teachers implement more differentiated instructional practices than upper secondary school teachers (Sánchez-Escobedo & Camelo, 2018; Schwab et al., 2019). Studies conducted by Liebner and Schmaltz (2021) as well as by Gilor and Katz (2018) revealed that there is a significant difference between the various types of initial teacher training within countries when it comes to pre-service teacher preparation for inclusion and differentiated practice. Likewise, this has been reported in the case of the development of pre-service teachers’ digital competences, which in several programs plays a minor role (Tiede, 2020). Hence, all considered, it seems reasonable to expect variations on teacher competences and characteristics resulting from the different educational pathways.

## Measuring teacher competence and characteristics: parents’ perceptions and evaluation

Clinton et al. (2016) state that “evaluating the performance and impact of teachers is an integral part of ensuring the quality and effectiveness of teaching” (p. 1). Additionally, evaluating teacher competence and characteristics can help identify and promote effective instructional practices that could serve as a means to support teacher professionalization (Taylor & Tyler, 2011). Moreover, it may facilitate implementing policies that support the retention of effective teachers (Goldhaber & Theobald, 2011). Current systems for teacher evaluation differ with regard to assessment frameworks and methods, using for instance value-added assessment results, formal lesson observations, and teacher portfolios and interviews, as well as peer and student ratings. However, certain countries continue to use value-added measures such as student test scores to determine teaching performance and effectiveness. To this fact, Master (2013) discusses that such measures provide little formative information to support teachers’ professional development.

Another source of information about schooling and teaching that has received less attention is the perspective of parents regarding teachers and the quality of educational provisions (Bethere & Pavitola, 2014; Master, 2013). Two main reasons for this have been discussed as arguments for the inclusion of parental evaluations in educational accountability systems. First, parental engagement has a significant effect on educational outcomes, and in order to enhance effective and meaningful parent participation, their voices should be taken into consideration (Bethere & Pavitola, 2014). Second, parents are key stakeholders in the educational system and should be included in teacher evaluation processes (Barrows et al., 2017; Clinton et al., 2016). According to the international review by Clinton et al. (2016) and the study by Master (2013), countries such as Australia, Hong Kong, South Korea, and the United States have started to implement parent measures as part of their teacher evaluation systems. Some of the aforementioned elements of professional teaching competence can be observed and therefore measured more objectively than others (van de Grift, 2014). For instance, it is easier for parents to see whether teachers use differentiated material for their children rather than formulating opinions on more complex and abstract aspects such as deciding on whether a teacher holds transmissive or constructivist beliefs.

Up to now, there has only been a limited amount of research making use of parent perspective for teacher evaluation; nonetheless, several insights can be extracted from their results. For instance, a study by Peterson et al. (2003) revealed that although parent evaluations tend to be positive, they still provide sufficient information to allow for differentiation between individual teachers. In addition, parent ratings appear to be moderately stable. Consistent with Peterson et al.’s study, Master (2013) also reported stable parent ratings of teacher performance over time. Additionally, the author found that parents’ and students’ perspectives were closely aligned, and that parent perspectives are also predictive of math teachers’ value-added performance ratings. On the other hand, studies by Chingos et al. (2012) and Favero and Meier (2013), conducted at the school level, have reported that parents accurately assess school outcomes.

### Parents’ concerns and judgements of teaching competence and characteristics during the COVID-19 homeschooling situation

Parental perception and evaluation, as thus far described, have been identified as a main educational quality indicator that can help to ensure quality improvement and reveal possible drawbacks of the education system (Bethere & Pavitola, 2014). A caring educational system in which parents, school staff and teachers support students’ basic psychological needs for autonomy, competence experience, and social integration as well as their learning process is very important for stable development (Eskreis-Winkler et al., 2014; Strayhorn, 2014). However, COVID-19 has decreased teachers’ role in active student learning time, and therefore has intensified parent control over their children’s learning processes (Fontenelle-Tereshchuk, 2021; Letzel et al., 2020; Pozas et al., 2021). This includes tasks like supporting, controlling, and reviewing completed learning tasks as well as monitoring their children’s motivational and volitional states and stress (Huber & Helm, 2020). Additionally, parents should support their children emotionally and provide technical prerequisites needed to make their attendance of online learning lessons possible (Huber et al., 2020). Schools as well as teachers should provide the technical, emotional, and cognitive resources to support children, in particular those who are rather socioeconomically disadvantaged, in order to compensate for a possible scissor effect between families (Helm et al., 2021). Even though the study by Huber et al. (2020) revealed that parents have praised and valued the work of teachers during this period of homeschooling, other studies report that parents considered there to be insufficient interaction during online classes (Zhou et al., 2020). Given the lack of interaction, the social isolation, and the negative consequences these might have on children and their mental health, they have been a major concern for parents (Fontenelle-Tereshchuk, 2021; Thorell et al., 2021). The fact that some parents in the study by Fontenelle-Tereshchuk (2021) noticed that teachers showed a tendency to rely heavily on pre-made materials as opposed to using technology in their lesson planning to construct authentic learning opportunities for students points at parents’ concerns about the digital competence of their children’s teachers. Moreover, findings from Thorell et al.’s (2021) study carried out across seven European countries indicated that parents found ERT to be of poor quality, with insufficient support from schools, and that contact with teachers was quite limited. Likewise, Letzel et al. (2020) reported parent concern about whether equal opportunities for all students could be guaranteed, that the students’ situations at home were not considered, and that teachers were not addressing the learning demands of students with special education needs. Taken together, current empirical research agrees with parents’ concerns about professional teaching competence: lack of feedback and communication, differentiated instruction, and low digital competence.

## The present study

The COVID-19 pandemic has presented several challenges for the educational system. Students had to learn from home, parents had to take over the roles of teachers, and teachers had to provide lessons digitally. This emergency situation required a close and stable collaboration between several stakeholders of the educational system in order to guarantee the continuation of students’ learning without major disadvantages. Many concerns were raised whether this situation could widen the gap between students due to different preconditions, equipment, or emotional support at home. This study takes parents’ perspectives into consideration and explores how parents evaluate their children’s teacher performance during the first lockdown in spring 2020. To investigate the parental evaluation of the teachers, the study focuses on the following research questions:

1. How do parents evaluate teachers during COVID-19 online learning in terms of domains of professional teaching competence and characteristics?
2. Do parental evaluations of teachers’ professional teaching competence and characteristics vary across educational pathways (primary and upper secondary schools)?
3. Is there a relationship between parental evaluations of teachers’ domains of professional teaching competence (i.e. digital competence, digital differentiation instruction, and feedback) and their perceptions of teacher characteristics?

## Method

### Participants and procedure

To examine the research questions, the present study follows a quantitative cross-survey design (Creswell, 2004) that makes use of data from parents (87% female), either the father or mother, of 721 children who participated in an online survey conducted during the initial four weeks of the first school shutdown in Germany in March and April 2020. From the total sample, 14.8% of the parents were born before 1970, 80.7% were born between 1971 and 1980, and 4.5% born after 1980. Further, the sample is composed of 41.7% of parents with children attending primary school (*n* = 301) and 58.3% of parents with children in upper secondary schools (*n* = 420). Lastly, the present sample comprises a total of 25.1% of parents holding a general or intermediate secondary education certificate, 13.5% parents with a general qualification certificate for university entrance, 10% parents with a diploma for vocational training, and 47% holding a university degree.

### Instruments

Besa et al., (2020) developed several scales in order to assess parents’ perceptions of the digital competence of their children’s teachers, their use of digital differentiated practices, and their personal characteristics. The scale items were based on a 5-point Likert scale ranging from 1 = *strongly disagree* to 5 = *strongly agree*. All scales used showed satisfactory to very good instrument quality. Parents’ perceptions of teachers’ digital competence were measured using 3 items (e.g. “My child’s teachers have the necessary skills to create digital learning materials”, α = 0.91). Four items were used to assess parents’ perceptions of teachers’ implementation of differentiated practices (e.g. “The digital learning materials take into account the individual abilities of my child”, α = 0.73). Lastly, in order to measure parents’ perceptions of specific teacher characteristics, the instruction “I usually experience my child’s teachers as …”, was presented followed by the single items of “motivated”, “flexible”, “appreciative”, “devoted”, “diligent”, “fair”, “helpful”, “professionally competent”, and “communicative”.

Lastly, parents’ perceptions concerning teacher feedback were measured using a scale from the Hamburg School Inspection (Pietsch et al., 2015). The scale consists of 5 items based on a 5-point Likert scale ranging from 1 = *strongly disagree* to 5 = *strongly agree* (e.g. “I feel well informed…about the goals and content of my child’s lessons”, α = 0.97).

### Data analysis

Statistical analyses were conducted in IBM SPSS Statistics 27. To explore the first research question, descriptive analyses and one sample *t*-test were performed. For the second research question, three one-way analyses of variance (ANOVA) were performed to explore differences between parents’ perspectives of teachers’ digital competence, differentiated instruction and feedback, and their educational pathways. Lastly, in order to examine the third research questions, Pearson correlations were calculated for each variable understudy.

## Results

### Parental evaluation of teachers’ professional competence and characteristics during homeschooling

Mean and standard deviation scores were calculated to determine parents’ evaluations of the digital competence of their children’s teachers, their use of differentiated instruction, feedback, and teacher characteristics. Given that the theoretical mean of the scales and single items was 3, as shown in Table 1, the total mean scores for all variables under study were significantly above the mean, thus indicating that parental evaluations of teachers’ competence and characteristics were in general fairly positive.

**Table 1:** Means, standard deviations, and one sample t-tests

	*M*	*SD*	df	t	*p*	Cohen’s d
1. Digital competence	3.33	1.15	702	7.61	< .001	1.15
2. Digital differentiated instruction	3.09	1.01	713	2.27	< .01	1.01
3. Feedback	3.57	1.05	720	14.66	< .001	1.05
4. Motivated	3.93	1.06	702	23.33	< .001	1.06
5. Flexible	3.47	1.15	697	10.86	< .001	1.15
6. Appreciative	4.03	1.05	694	25.89	< .001	1.05
7. Devoted	3.94	1.06	699	23.41	< .001	1.06
8. Diligent	3.90	1.04	679	22.53	< .001	1.04
9. Fair	3.88	0.98	656	23.00	< .001	0.98
10. Helpful	4.14	0.97	706	31.39	< .001	0.97
11. Professionally Competent	4.25	0.85	700	38.82	< .001	0.85
12. Communicative	3.85	1.07	712	21.25	< .001	1.07

### Differences across educational pathways

In order to examine whether parental evaluations differ across educational pathways (primary and upper secondary schools), three one-way ANOVA for teachers’ digital competence, digital differentiated instruction, and feedback were conducted. For parent evaluation of teachers’ digital competence, a significant main effect of educational pathways, *F*(1,702) = 16.92, *p* < .001, *η* ^*2*^ = 0.02, revealed that parents perceive that teachers’ digital competence varies across the two educational pathways. In detail, parents with children attending primary schools (*M* = 3.55; *SD* = 1.29) rated a higher level of digital competence than parents whose children attend upper secondary schools (*M* = 3.19; *SD* = 1.03). Concerning parents’ evaluation of teachers’ digital differentiated instruction, the analysis yielded a significant main effect of educational pathways, *F*(1,713) = 10.76, *p* < .001, *η* ^*2*^ = 0.02. Results indicate that parents whose children attend primary schools (*M* = 3.23; *SD* = 1.13) rated teachers’ digital differentiated instruction significantly higher than did parents with children registered in upper secondary schools (*M* = 2.98; *SD* = 0.91). Lastly, a significant main effect of educational pathways was found for the variable of parents’ evaluation of teacher feedback, *F*(1,720) = 52.65, *p* < .001, *η* ^*2*^ = 0.07. In detail, parents whose children attend primary schools (*M* = 3.90; *SD* = 1.00) rated teachers’ feedback instruction significantly higher than did parents with children registered in upper secondary schools (*M* = 3.34; *SD* = 1.03).

### Correlations between teachers’ digital competences, digital use of differentiated instruction, feedback, and their personal characteristics

Pearson correlations were calculated for each variable in the study. As indicated in Table 2, positive correlations were found between parental evaluations of teachers’ digital competence, digital differentiated instruction, feedback, and all of the aforementioned single items encompassing teachers’ characteristics. In particular, it is important to highlight that the strongest correlations were found between parents’ evaluations of teachers’ feedback and how motivated, appreciative, and devoted teachers are perceived to be.

**Table 2:** Pearson correlations of all variables

	1	2	3	4	5	6	7	8	9	10	11	12
1. Digital competence	-											
2. Digital differentiated instruction	.58**	-										
3. Feedback	.47**	.50**	-									
4. Motivated	.57**	.46**	.61**	-								
5. Flexible	.64**	.49**	.55**	.68**	-							
6. Appreciative	.44**	.44**	.63**	.73**	.62**	-						
7. Devoted	.52**	.48**	.62**	.72**	.68**	.76**	-					
8. Diligent	.57**	.49**	.58**	.78**	.67**	.69**	.68**	-				
9. Fair	.48**	.47**	.55**	.66**	.63**	.70**	.69**	.61**	-			
10. Helpful	.50**	.44**	.55**	.72**	.66**	.72**	.73**	.68**	.68**	-		
11. Professionally Competent	.45**	.43**	.55**	.62**	.55**	.65**	.63**	.60**	.62**	.59**	-	
12. Communicative	.54**	.45**	.60**	.69**	.67**	.66**	.72**	.62**	.56**	.68**	.56**	-

## Discussion

Given the unprecedented impact that COVID-19 has had on the educational field worldwide, information on teacher competence and effectiveness during ERT has become necessary to mend the current repercussions as well as to develop teaching for after the pandemic. Parental evaluation, although only seldom explored in research, has nevertheless been included in different teacher evaluation systems (Clinton et al., 2016; Master, 2013). Given that strong parental participation and engagement were needed in order to continue education from home, and that parents themselves took over teachers’ roles (Letzel et al., 2020), parents have become an important source of information. With this in mind, the present study sought to explore parental evaluations of teachers’ competence and characteristics during the first period of COVID-19 school closures in Germany.

### Parental evaluations of teachers’ professional competence and characteristics

Descriptive results revealed that, in general, parents evaluate teachers highly positively in all teacher competence domains as well as those regarding teacher characteristics. Such findings are underscored by the large effect sizes across all variables. Additionally, such results are consistent with previous studies that have shown that parents tend to evaluate teachers in a positive light (Master, 2013). In particular, parental evaluations concerning teacher characteristics are also consistent with Helm et al.’s (2021) systematic overview of empirical research on teaching and learning during the COVID-19 school closures. For instance, Helm et al.’s (2021) overview reported that parents in general considered teachers to be highly motivated (Dreer et al., 2020). However, when it came to teachers’ digital competence, their use of differentiated instruction, and feedback, this study’s results were quite different to what is discussed in Helm et al.’s (2021) systematic overview. According to the authors, most surveys revealed that parents consider teachers’ lack of digital competences to be the biggest problem during ERT (Huber et al., 2020; Fontenelle-Tereshchuk, 2021) discusses that parents raised concerns about these limited digital competences because teachers mostly used a range of materials they made or prepared themselves as opposed to adopting digital tools and instruments. With regard to differentiated instruction, a recent systematic review of literature conducted by Bond (2020) indicates that, in general, there was a lack of differentiation in addressing students’ learning needs – special education and otherwise - during ERT. Similar results were also found in the studies by Helm et al. (2021) and Letzel et al. (2020), in which the vast majority of parental surveys reported that teachers only rarely addressed their students’ individual learning needs by adapting their instruction, or did not do so at all. The same picture can be drawn for teacher feedback: the majority of research reports that parents perceived a lack of feedback during the COVID-19 school closures (Steinmayr et al., 2021; Zhao et al., 2020). A possible explanation for the contradictory findings between this study and other COVID-19 parental perception research could be a result of the present study’s sample. The sample is composed predominantly of parents with ahigh level of educational attainment. For example, according to Wößmann et al, (2020), parents with such academic qualifications perceived more teacher feedback compared to parents with lower educational attainment. Moreover, given that the sample comprises mostly parents and/or families with higher socioeconomic status, it stands to reason that they also had better access to digital tools and resources.

### Teacher competence and characteristics: differences across educational pathways

As discussed in the theoretical section, the expectation that educational pathways could explain differences in teacher competence and characteristics were supported by the results from this study’s one-way ANOVA analyses. With regard to parental evaluation of teachers’ digital competences, our findings reveal that primary school teachers are rated more highly than teachers in upper secondary schools. Current research has provided heterogeneous results: several studies have indicated that teacher competence correlates with higher school grades or classes as well as educational pathways (Forsa, 2020), whereas other studies have indicated the opposite (Schwab et al., 2020). This mixed evidence most probably originates from the different instruments used to measure the teachers’ digital competence. Nonetheless, it is also important to highlight that the integration of digital media preparation in initial German teacher training programs varies significantly across educational pathways and universities (Tiede, 2020). Thus, it would be beneficial to conduct further research to explore whether such differences stem from teacher preparations programs, or particular experience with primary school.

Regarding parental evaluations of teacher differentiation during ERT, results from the one-way ANOVA revealed significant variations between teachers in primary and upper secondary schools. In detail, parents rated higher levels of differentiation being performed by teachers in primary schools compared to those of upper secondary school teachers. Such results are in line with research comparing differentiation use between teachers of both educational pathways (Deunk et al., 2015; Pearce et al., 2010);Schwab et al. (2019) point out that “the curriculum and teaching practices in secondary classrooms are markedly different from practices in primary classrooms” (p. 4). When it comes to teachers’ differentiation during ERT, Schwab et al. (2020) and Feistritzer et al. (2020) reported a similar trend; that is, that upper secondary teachers adapted their instruction according to their students individual learning needs less often.

Furthermore, the one-way ANOVA analysis revealed significant variations between parent evaluations of teachers’ feedback between primary upper secondary schools. Thus, such results indicate mainly a difference between the educational pathways. Research on teacher support, such as feedback, has indicated that students perceive a decline from primary to secondary school (Byberg & Tybring, 2004). However, Bru et al. (2010) argue that this could be resultant of age rather than the education pathway itself. When observing current research during the COVID-19 closures, studies thus report no differences between the educational pathways (Helm et al., 2021). In this line, further research needs to be conducted in order to explore such findings in more detail.

### Associations between teachers’ competence and characteristics

Lastly, the correlation analyses indicate that there is a strong and positive relationship between how parents evaluate teaching competence and characteristics as well as teachers’ personal characteristics. Even though in the present study domains of teacher characteristics were statistically linked through means of a correlational analysis and not through sophisticated modelling, the findings obtained are still consistent with numerous previous studies that have empirically tested and validated the interrelations between the teaching competence domains, such as classroom management, cognitive activation, competence, values, and beliefs, among others (e.g. Hamre et al., 2013; Praetorius et al., 2018; Maulana et al., 2017; Maulana et al., 2020). This is in general an important finding. As discussed within the theoretical background, teaching quality in face-to-face schooling may not directly translate into effective instructional behaviors within an online teaching and learning environment (Rice & Dawly, 2009). Thus, the findings provide an initial glimpse into the interrelations between the elements of teaching competence and characteristics that are also crucial for ERT. With this background, the study results call for further research to explore teaching quality within distance learning.

## Limitations

Several limitations must be considered. First, several scales used within the study are not yet standardized or validated. However, given the extraordinary nature of the COVID-19 pandemic school closures, previously well-established instruments might not be appropriate to explore such an unprecedented situation, though it is nevertheless necessary for the study’s instruments to be further explored and validated. Moreover, the instruments used assessed parental evaluations of their children’s teachers, and not a particular educator (e.g. a homeroom teacher or subject-specific teacher). Given that parents evaluated their children’s teachers in general, the findings do not allow to examine in detail profound differences between single teachers. A second limitation is that the sample comprises mostly parents with a higher level of academic attainment. Thus, the findings obtained may not be representative of or generalizable with other samples. Moreover, this study did not consider parents with children in lower secondary schools, which are different from upper secondary schools. Taken together, further research should strive to balance the samples with participants who have adhered to secondary school tracks such as comprehensive schools or intermediate schools, and by including special education (Scheer & Laubenstein, 2021). This is crucial, as most research investigating parental experiences of ERT reported a lack of instructional support for their children’s special education needs (Thorell et al., 2020) and indicates additional limitations, as data on parents’ and their families’ socioeconomic situations as well as their migration backgrounds were not collected for this study. Wildemann and Hosenfeld (2020) explain, for instance, that families with lower socioeconomic status faced greater challenges with ERT or homeschooling. Thus, the results of this study must be considered with caution. This limitation stands also for the fact that data on student achievement was not collected, and thus, not controlled for; empirical studies have indicated that teacher competence has an impact on students’ achievement (Maulana et al., 2017). Hence, the findings of this study do not allow to draw conclusions on the effect of teaching competence and characteristics on students’ achievement outcomes. Third, this study follows a cross-sectional design, and thus uses data collected only during the first COVID-19 school closure in Germany. Given the numerous changes within schools during pandemic closures, it is plausible that such parental evaluations could have been affected throughout the past year (Schmidt et al., 2021). Fourth, the data collected stems from two different states in Germany. Though all schools in the country were closed during the period in which the study took place, there could be significant variations on how each school, or more specifically, each teacher, managed ERT. In other words, there might be important differences across such factors as resources allocated, how teachers used resources at hand, and how parents experienced and perceived education in the first place. Lastly, as discussed by van de Grift (2014), the elements of professional teaching competence can be perceived and assessed quite differently. Therefore, future research should not only involve parents, but also stakeholders such as teachers and students, and should make use of different data sources, such as class observations and interviews (Fauth et al., 2020).

## Practical implications and conclusion

With the COVID-19 pandemic, parental engagement, participation, and commitment were necessary in order for education to continue. As a result, parents took over the roles of teachers, which gave them a new perspective on how teaching and learning are developed. In particular, given that parents “learned” in far greater detail what a teacher’s job entails, it could be assumed that parents’ current evaluations reflect a more realistic picture than they may have previously, as they experienced the role of a teacher firsthand. In this vein, it would be important for further research to explore in-depth parental changes in perceptions of teacher competence and characteristics, for instance, through interviews. This could shed light on a better understanding of teacher effectiveness during the pandemic. Moreover, the present paper calls for parents to be considered as key stakeholders within educational evaluation systems even after the pandemic. Thus, it is important to bridge the gap between parents and teacher evaluation systems in order to ensure meaningful and quality education for students, not only during the current pandemic challenges, but in the future to come.
